# From the bench to the bedside: Stimulating science from around the globe

**DOI:** 10.4103/2152-7806.69377

**Published:** 2010-09-16

**Authors:** Jason S. Hauptman

**Affiliations:** Intellectual and Developmental Disabilities Center and Department of Neurosurgery, Geffen School of Medicine at UCLA, Los Angeles, CA, USA

## IN THIS UPDATE

- Motor cortex stimulation results in amelioration of motor deficits from unilateral corticospinal tract (CST) injury
- Astrocytes discovered to play a major role in breathing
- Detecting microseizures using implantable microelectrodes provides insight into the epileptic brain

## REPAIRING THE MOTOR SYSTEM AFTER INJURY:MOTOR CORTEX STIMULATION MAY BE THE ANSWER:[1]

### Key points

1. Electrical stimulation has been shown to have beneficial neurotrophic effects *in vitro* and *in vivo*.
2. Epidural motor cortex stimulation of the intact hemisphere in a unilateral model of CST injury results in improvement of motor deficits as well as axon outgrowth in the damaged fiber tract.
3. Motor cortex stimulation is already an approved method of treating deafferentation pain syndromes.

The CST consists of neurons responsible for voluntary movement, with connections that span the motor cortex down to the alpha motor neurons that innervate specific muscle groups. Because trauma to the CST occurs frequently during brain and spinal cord injury and results in devastating motor impairments, the authors explore a technique that has been proposed to help restore CST connections: chronic CST electrical stimulation. Prior work has shown that electrical stimulation can result in increased synapse formation during axon development, and that when CST axons are not stimulated the result is decreased CST connections and motor impairment. Furthermore, it is suggested that stimulation of the intact (uninjured) motor fibers may restore motor function to the injured CST. In this study, Carmel *et al*. used a model of unilateral motor cortex injury to see if electrical stimulation of the intact (uninjured) side can result in restoration of motor deficits.

After lesioning one of the pyramids in a rat model, the authors performed epidural electrical stimulation of the motor (M1) cortex of the opposite (uninjured) hemisphere. Stimulation was performed using stainless steel epidural electrodes implanted stereotactically, started 6 hours after injury, and continued until day 10 following injury. They found that the stimulated, injured rats experienced complete resolution of motor deficits by 30 days following injury. This is particularly interesting because the most remarkable gains in motor function occurred after stimulation ceased (at about post-injury day 20), with the beneficial effects continuing as long as they were tested (30 days total following injury). This was not unique to the motor cortex (even when the authors directly stimulated the intact pyramid, the same effects were seen). Furthermore, detailed analysis of motor performance suggested that this improvement in motor function was most likely due to restoration of normal motor control rather than compensatory mechanisms or alternative movement strategies.

Because prior work has shown that stimulation of the intact pyramid results in neurite outgrowth of the CST in the cervical spinal cord, the authors wanted to see if motor cortex stimulation would result in similar neurotrophic effects. After day 30 following the motor analysis of the stimulated and unstimulated animals, rats were sacrificed and axons were labeled to see if growth in the injured CST occurred. They found that stimulation resulted in significant CST axon outgrowth within the normal dorsoventral projections of CST neurons.

This is a very exciting study since intact motor cortex stimulation is extremely technically feasible and safe. Of course, more work is necessary to understand the mechanisms by which axon outgrowth and motor improvements occur. Furthermore, it is unclear if this can be directly translated to humans. That being said, motor cortex stimulation is already an acceptable therapy for patients with deafferentation pain syndromes. Experimental noninvasive methods of motor cortex stimulation, such as focused ultrasound or magnetic stimulation, may also be applicable. This could provide an exciting future for those with motor deficits resulting from cerebrovascular disease or CNS trauma.

## ASTROCYTES FOUND TO HAVE A MAJOR ROLE IN PHYSIOLOGICAL REFLEX:[2]

### Key points

1. Astrocytes demonstrate changes in excitability by fluctuating their internal concentrations of calcium. These calcium changes are transmitted to adjacent astrocytes through gap junctions and the vesicular release of neurotransmitters such as ATP.
2. Astrocytes of the ventral medulla play a crucial role in the modulation of breathing, particularly, the stimulus to breath in the presence of acidosis. This is accomplished likely through the release of ATP, which activates neurons responsible for the actual breathing reflex.

Traditional neuroscience teaching suggests that astrocytes function primarily to support neuronal function, particularly by creating a friendly metabolic environment for neurons to operate optimally. Until recently, little work has been done to show whether astrocytic modulation can play a demonstrable role in behavior. This is in part because astrocytes do not fire action potentials. They are not inert, however—astrocytes do demonstrate changes in calcium concentrations that are thought to play a role in their function. When an astrocyte is “excited”, it experiences a transient increase in intracellular calcium released from intracellular stores. This calcium excitation can occur in response to a variety of metabolic stimuli through activation of metabotropic G-proteins at the cell surface. These proteins initiate a signaling cascade, resulting in the internal release of calcium into the astrocyte’s cytoplasm. Furthermore, this increase in calcium can spread to neighboring cells via gap junctions, producing a spreading calcium wave that near-synchronously excites adjacent astrocytes. In this study, Gourine *et al*., used cutting-edge techniques of neuromodulation to show that astrocytes play a critical role in the control of a fundamental physiological reflex: breathing.

First, the authors demonstrated that hypoventilation and resultant acidosis in anesthetized rats resulted in an immediate increase in intracellular calcium of astrocytes located within the ventral medulla (VS). This calcium excitation response to acidification was replicated *in vitro* using brain slices and cell cultures. To make sure that this change was not due to excitation of adjacent neurons in the same region, drugs were applied to block neuron action potentials and synaptic excitation. Even in the presence of these drugs, the acidification-induced calcium excitation of astrocytes persisted. Prior work suggests that this calcium excitation is likely mediated by the presence of ATP binding to cell surface receptors. ATP is a source for cellular energy as well as a neuromodulating chemical. In fact, when ATP actions are blocked, the acidification-induced calcium excitation response is reduced.

This calcium response in astrocytes spreads among adjacent astrocytes. This occurs through the presence of gap junctions (channels between astrocytes that are big enough to allow the passage of calcium ions) as well as the vesicular release of ATP (similar to the synaptic release of neurotransmitters by neurons). The actions of ATP are not limited to astrocytes, however. Converging lines of evidence suggest that ATP also stimulates the adjacent neurons in the VS, resulting in stimulation of breathing. In fact, when ATP signaling is blocked, these neurons do not stimulate the breathing reflex in the presence of acidification.

Using a combination of genetic techniques, the authors engineered rats with VS astrocytes that undergo calcium excitation in the presence of exposed light from a fiberoptic probe. These rats were anesthetized, vagatomied, and placed on a ventilator. When these astrocytes were “activated” with light, increases in respiratory activity were observed. Thus, calcium excitation in these astrocyte populations results in a directly observable behavior. This is an extremely exciting observation since it calls into question assumptions about complex behaviors being due to the sole actions of specialized neurons. Astrocytes may no longer just play a supporting role to neurons, but instead are being launched right into center stage.

## NEEDLES IN A HAYSTACK:THE PRESENCE OF MICROSEIZURES IN THE EPILEPTIC BRAIN.[3]

### Key points

1. Implantable electroencephalography (EEG) microelectrodes provide improved spatial and temporal resolution for studying epileptic events in small volumes of brain tissue (microdomains).
2. Epileptic brain demonstrates greater frequency and duration of microseizure events compared to control brains. Even though these events occur in a wide range of brain areas, the frequency of microseizure events is doubled in the ictal onset zone.
3. Microseizure events appear to be characteristic of epileptic brain and may provide insight into more precise definitions of seizure-generating brain regions.

The surgical treatment of epilepsy relies on proper identification of brain areas responsible for the generation of seizures. Current modalities employed for finding the surgical target include intracranial EEG, which suffers from limitations of spatial and temporal resolution. Newer techniques that record with higher bandwidth (meaning a greater range of frequencies) and greater spatial resolution (on the scale of cubic millimeters of tissue), including implantable microelectrodes, have identified smaller brain regions (called microdomains) with seizure-like discharges that may play a role in identification of seizure generators. In this study, the authors used implantable macro- and microelectrodes in patients with epilepsy to further evaluate these microdomains.

After reviewing over 7000 candidate microseizure events from the research microelectrodes, the authors identified electrical events categorized as microseizure activity that was spatially attributed to a single microelectrode. Patients with epilepsy tended to have more and longer microseizure activity compared to controls. In patients with partial epilepsy in particular, microseizures occurred in non-contiguous and highly localized microdomains. This activity did not evolve like macroelectrode seizures, instead remaining stable and only detectable by the single microelectrode in that region. Interestingly, in almost 20% of seizures detected by macroelectrodes, this microseizure activity actually preceded the macroelectrode seizure and continued throughout its evolution. Microseizure activity was seen in a wide range in brain regions, both inside and outside the ictal onset zone (the brain area where seizures begin). However, microseizure events within the ictal onset zone occurred in more than double the frequency of regions outside of the ictal onset zone.

From these data, the authors conclude that the epileptic brain consists of isolated microdomain regions that exhibit this microseizure activity. Furthermore, microseizure activity occurs at a higher rate within the ictal onset zone, suggesting that these events may be characteristic of epileptic regions. It is unclear, however, whether these isolated microdomains are a cause of or the result of epileptic activity. It is possible that these microdomains are the pathological signature of abnormal activity that led to the development of epilepsy. It is also possible, however, that this activity is a result of brain pathology secondary to disruption of normal networks from seizure activity. That may explain, for example, the presence of microseizures in tissue outside of the ictal onset zone.

The authors propose that microseizure activity within these microdomains may spread to other areas and eventually cause macroelectrode seizures. This may be enhanced by conditions that lower the seizure threshold. It will be interesting to see future work dedicated to understanding the meaning of these microseizure events. Are they neuronal? Can they be contaminated by glioneuronal networks? Is there a role of subthreshold membrane oscillations within neurons? The high spatial resolution achieved in this study and the convincing association of microseizure events with epileptic brain suggest that microelectrode seizure detection may play a larger role in the precise definition of seizure-generating regions of brain. Their interesting observations open the door to future electrophysiological studies examining the nature of microdomains and their function in seizure formation.
